# A Novel Method to Assess Subject‐Specific Architecture of the Achilles Tendon In Vivo in Humans

**DOI:** 10.1111/sms.70042

**Published:** 2025-03-26

**Authors:** Taija Finni, Raad Khair, Jason R. Franz, Maria Sukanen, Neil Cronin, Stephanie Cone

**Affiliations:** ^1^ Faculty of Sport and Health Sciences, Neuromuscular Research Center University of Jyväskylä Jyväskylä Finland; ^2^ Joint Department of Biomedical Engineering University of North Carolina Chapel Hill and North Carolina State University Chapel Hill North Carolina USA; ^3^ School of Education & Science University of Gloucestershire Gloucestershire UK; ^4^ Department of Biomedical Engineering University of Delaware Delaware USA

**Keywords:** Achilles tendon, anatomy, electrical stimulation, MRI, structure, subtendons, ultrasonography

## Abstract

The Achilles tendon (AT) comprises three subtendons whose relative locations, and respective lines of action, vary individually. This study was aimed to demonstrate the efficacy of a novel method, combining Ultrasound and electrical STIMulation (USTIM), to identify the in vivo location of individual subtendons in cross‐sections of the AT. We individually stimulated the triceps surae muscle heads and imaged localized tissue movement on a transverse plane 1 cm proximal to the calcaneus using B‐mode ultrasonography. Movement induced by muscle stimulation was presumed to arise from movement in the respective subtendon. Frame‐by‐frame changes in grayscale values were analyzed to detect localized tissue movement, establishing the three subtendon locations. From 12 successfully assessed legs, we found test–retest reliability to be excellent (ICC = 0.93, *N* = 3), and intra‐ and inter‐rater reliability to be good for the subtendon centroid locations (ICC > 0.77, *N* = 12). Reliability for identifying the subtendon area was good for test–retest (ICC = 0.77) and intra‐rater assessments (ICC > 0.70) but moderate between raters (ICC = 0.53). Subtendon centroid locations assessed using USTIM showed a strong association (*N* = 2; *r* ^2^= 0.80, *p* < 0.001) with those identified via the high‐field MRI method established by Cone et al. Fitting with prior literature, the majority of (83%) tendons were identified as low twist type I. The novel USTIM method can identify in vivo locations of the three subtendons within a cross‐section of AT with moderate to excellent reliability. This method could be used to unravel the intricacies of structure–function relationships in the AT, with potential clinical benefits for treatment of patients with AT injuries.

## 1 Introduction

Currently, our knowledge of Achilles tendon (AT) architecture is derived primarily from cadaver studies [1, 2, 3], and there is a lack of in vivo methods for study in humans. The AT is particularly complex, with subtendons arising from the uniarticular soleus (SOL) and biarticular medial (MG) and lateral gastrocnemius (LG) muscles, which merge and twist while descending from their respective muscle‐tendon junctions to the calcaneal insertion [1]. These subtendons have unique mechanical properties, making the AT susceptible to regionally variable shear stresses and strains [4] while providing redundancy to retain the function of the triceps surae unit in cases of injury [5]. While the AT is large and functionally important for locomotion [6], it is also susceptible to injuries including tendinopathies and rupture [7, 8].

To understand the mechanics and function of the AT in health and disease, knowledge of the underlying structure is vital. A growing number of studies have shown that the anatomy of the three AT subtendons varies individually in relative size and axial position [1, 2, 3, 9]. Based primarily on cadaveric studies, researchers have been forced to assume the relative location of the subtendons [10, 11]. Only very recently have some attempts been made to objectively identify subtendon locations in vivo. Cone et al. [12] used high‐field (7 T) magnetic resonance imaging (MRI) to describe the three‐dimensional (3D) AT structure in vivo in healthy young adults, expanding prior findings on subtendon structure throughout the length of the free tendon. While high‐field MRI can provide a comprehensive reconstruction of the tendon, more accessible and low‐cost methods should be developed for everyday assessments. Some research groups have used percutaneous electrical stimulation when trying to identify subtendon locations [11, 13, 14, 15]. These studies used the assumption that the force induced by selective stimulation in one muscle would be transmitted to its respective subtendon. While some lateral force transmission may occur at the subtendons and muscular levels [16, 17], the initial and the most prominent displacements are expected at the subtendon corresponding to the stimulated muscle. The subsequent displacement of localized tendon tissue is then visualized by ultrasonography (US) and the displacement pattern is used to infer subtendon locations. Khair et al. [8], Lecompte et al. [18], Lehr et al. [15] visualized the AT in the sagittal plane, allowing tracking of longitudinal movement of the tendon in a two‐dimensional view that does not necessarily contain information about all three subtendons. On the other hand, Klaiber et al. [14] visualized the transverse section where all three subtendons can be localized on a given plane. Theoretically, if stimulation of a muscle induces displacement in the respective subtendon, and this movement can be identified in a cross‐sectional plane, for example, due to torsion, the locations of each subtendon could be identified within an axial cross‐section of the AT.

Therefore, in this paper we introduce and demonstrate the efficacy of a new method—USTIM (i.e., Ultrasound and electrical STIMulation) ‐ to identify the in vivo location of individual subtendons from a cross‐section of the human AT. After pilot testing for method development, we assessed 14 healthy adults and examined the extent to which the USTIM method provides individual structural information of AT that resembles those reported in cadaver studies. Finally, we compared AT subtendon locations in two individuals assessed using both USTIM and the high‐field MRI method reported previously by Cone et al. [10].

## 2 Methods

### 2.1 Participants

Healthy adults aged 18–55 years were recruited, and 8 females and 6 males [mean (standard deviation) age 30 (5) years, height 172 (9) cm, body mass 73 (14) kg] gave informed written consent to participate. Exclusion criteria were a history of lower extremity musculoskeletal injuries or conditions limiting physical activity in the past 6 months. Of the 14 participants, two had both right and left legs assessed, and three had their right leg assessed twice. Two participants had their right AT imaged with high‐field MRI (details below). Laboratory assessments were done at the University of Jyväskylä, and MR images were collected at the University of North Carolina Chapel Hill (UNC‐CH). The study received approval from the Human Research Ethics Committee of the University of Jyväskylä on 21.12.2022 (1351/13.00.04.00/2022). The MR images were collected following informed consent as per the UNC‐CH Biomedical Sciences Institutional Review Board (IRB # 21‐0059).

### 2.2 Protocol

Participants lay in a prone position on a plinth with the ankle joint at 90°. Then, the most distal muscle‐tendon junction points of MG, LG, and soleus and the most proximal point of the calcaneus were scanned using ultrasound. These points were marked on the skin, and the distance between the calcaneal mark and the muscle‐tendon junction of the marked muscles was measured with a measuring tape following the curvature of the leg [19]. Based on protocol piloting, we selected a 90° ankle joint angle to facilitate a non‐zero baseline level of tendon tension and thus more reliable transmission of electrical stimulation to tendon tissue displacement compared to more plantarflexed joint postures.

**FIGURE 1:**
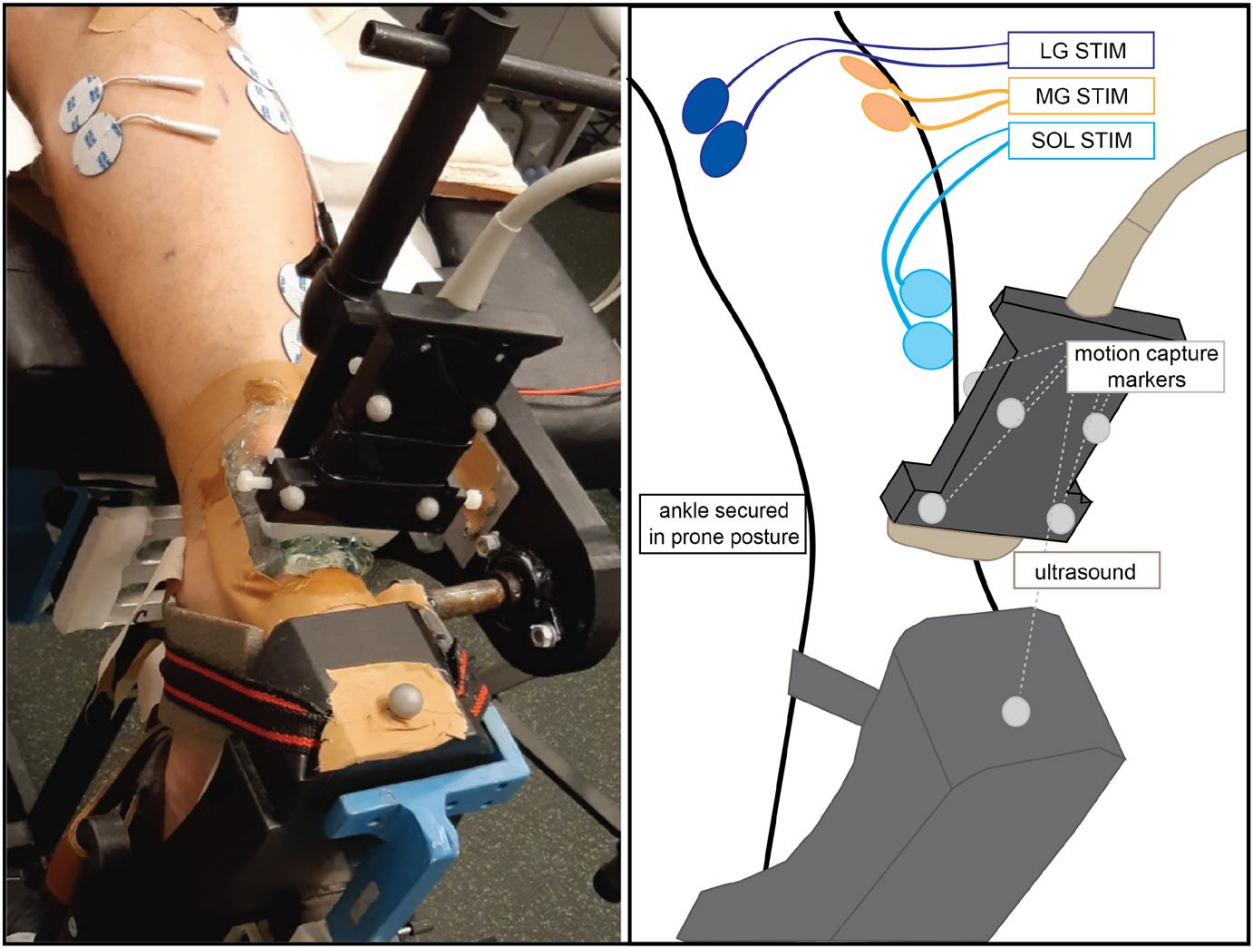
The experimental setup is pictured (left) and with key components diagrammed (right). Participants lay prone with the ankle joint at 90°, stimulating electrodes attached to MG, LG, and SOL muscles. The ultrasound transducer was mounted into a probe holder that was fixed approximately 1 cm proximal to the most proximal tip of the calcaneus. The current study did not utilize the motion capture markers.

Following Khair et al. [13], we placed 32‐mm round stimulating electrodes (Polar electrodes, Niva Medical Oy) with a 1 cm interelectrode distance over the muscle bellies of the SOL, MG, and LG. Electrode locations were identified using the US and placed over the thickest part of each muscle belly. For SOL, the electrodes were placed on the lateral side between the muscle‐tendon junctions of SOL and LG (Figure 1).

A constant‐current stimulator (DS7AH; Digitimer, UK) was used to elicit 1 ms single pulses to find a motor threshold for each stimulation location. Motor threshold was defined as the lowest stimulation intensity that induced muscle contraction that could be visually detected from the skin surface. Tendon displacement was imaged using the US during the application of a 0.7 s pulse train (1 ms pulse with 100 Hz frequency) to different muscles. First, motor threshold intensity (100 ± 32 mA for SOL, 107 ± 50 mA for LG, and 80 ± 42 mA for MG) was used to elicit the trains and increased by 5–15 mA to elicit a clearly visible displacement in the US image. Muscle contraction was visually monitored to ensure no cross talk between neighboring muscles. Participants were always asked if the intensity could be further increased, but low intensity was preferred for highly targeted stimulation and patient comfort. MG, LG, and SOL were stimulated in random order. Plantarflexion force and stimulation signals were sampled at 1000 Hz via a 16‐bit A/D board (Power 1401, Cambridge Electronic Design, Cambridge, UK).

### 2.3 Ultrasound Assessments

B‐mode US images were acquired using a 38‐mm linear transducer (5–18 MHz, SL18‐5, Aixplorer Supersonic Imagine, v. 12.3.1 Aix‐en‐Provence, France) transversely through the AT (Figure 1). The scanning location was approximately 1 cm proximal to the most proximal calcaneus echo signal. A relatively distal location was chosen to allow comparison of resulting subtendon locations to those previously assessed at the calcaneal AT footprint [2]. B‐mode videos (720 × 540) were sampled at 75 frames/s for ~3–6 s and exported with full frame rate as DICOM sequences and converted to AVI using a custom‐made MATLAB script.

### 2.4 Identification of Subtendon Locations

The analysis process of the cross‐sectional videos was motivated by the premise that regions of tendon tissue with the earliest displacements are those anatomically associated with the stimulated muscle. This assumed that the force produced by a given muscle is readily transmitted in series to its respective subtendon. The force is expected to cause displacement within AT in longitudinal and transverse directions, which can be identified as changes in grayscale intensity in the ultrasound images.

**FIGURE 2:**
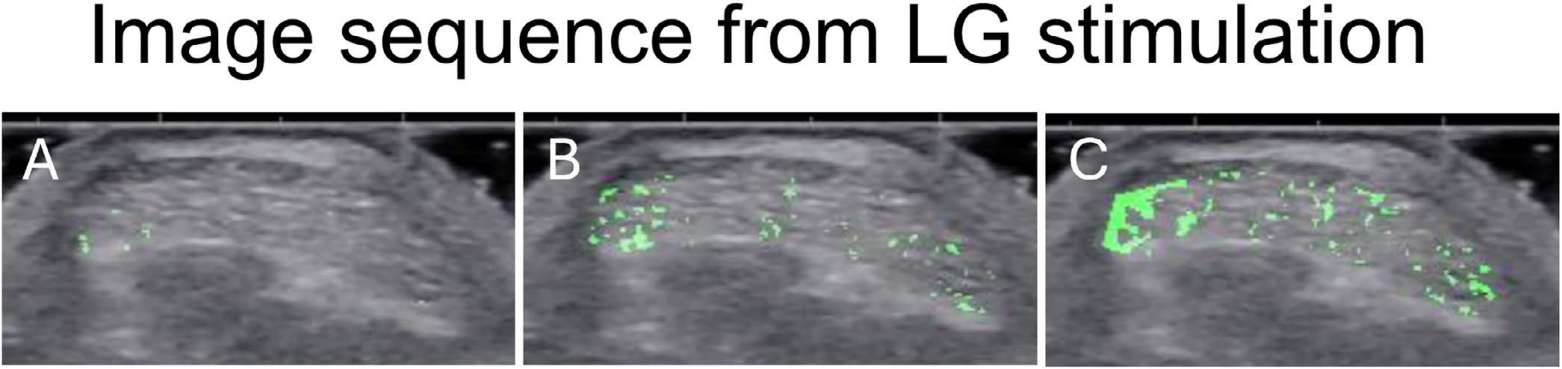
Example of analyzed image sequence where LG was stimulated. Three consecutive images (A–C), where echo‐intensity changes have been identified and masked with green are shown. The first movement in response to LG stimulation occurs in the lateral region (A). In the second image, 13 ms later, a larger lateral area is identified, but other locations also show displacement, which may be due to lateral force transmission (B). In the third image, 26 ms after the first detected movement, most movement is identified laterally, but also other locations show displacement (C).

**FIGURE 3:**
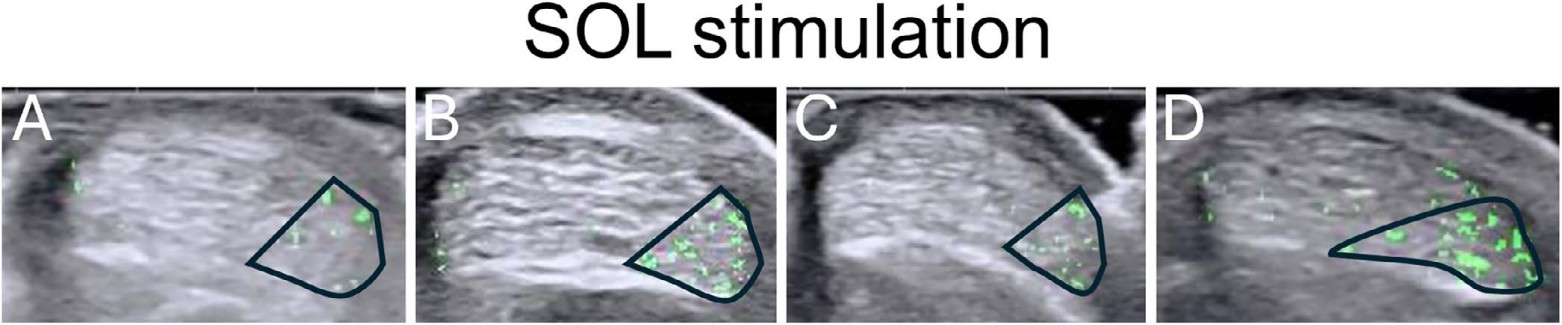
Examples of masks resulting from SOL stimulation in four different individuals (A–D). In all these cases, SOL subtendon was located medially (on the right‐hand side). The identified subtendon CSAs are outlined. In all examples but C there are also green areas outside the identified SOL region. These areas were identified to belong to other subtendons based on the decision‐making criteria.

Custom scripts were developed in MATLAB (R2023b, MathWorks Inc., Natick, MA, USA). For each participant, the AT cross‐sectional area (CSA) was first manually drawn as the region of interest (ROI) from a frame of the tendon at rest. The contraction caused only negligible changes (few degrees of rotation) to tendon cross‐sections, thus, the ROI defined at rest could be used for the entire sequence. The ROI was hand‐drawn using a polygon tool on the deep border of the AT. Then, the pixels within the ROI were converted to grayscale (0–255; zero represents the darkest shade, 255 the lightest), and the difference in grayscale intensity between successive frames was computed (Figure 2). After analyzed image sequences from each stimulation condition were reviewed visually, subtendon regions were manually outlined onto the AT cross‐section based on the between‐frame displacement information and the decision‐making strategy explained below (Figures 2 and 3). Appendix S1 contains example analysis from one participant with raw data shown in supplement videos S1–, S3.

For a given pixel, if the difference in grayscale intensity between successive frames was higher than a predefined initial threshold (6.0), the pixel was highlighted in green to denote displacement in this region. This initial threshold could be adapted per subject to find an optimal level, as the change in echo intensity can depend on the tissue quality and magnitude of movement occurring in response to stimulation. The goal of the analysis was to allow detection of distinct regions that move immediately after stimulation, thus revealing the subtendon location. The threshold was optimized to eliminate instances of multi‐region detection, optimizing for the excitation of a single subtendon with each muscle stimulation. Thresholds between 3 and 11 (referring to echo‐intensity change on the scale 0–255) were used in this study.

Two raters analyzed all trials independently and used the analysis and decision‐making strategy assuming that regions of tendon tissue with the earliest and most prominent displacements are those anatomically associated with the stimulated muscle in the following order of priority: (1) check the multiple trials recorded and start with the lowest stimulation intensity that shows visual movement within the tendon, (2) use low threshold initially and increase it if distinct areas do not become visible, (3) if the same area moves when stimulating different muscles, use the lowest stimulation intensity and threshold to decide the locations and use exclusion strategy for other subtendons after that, (4) visually evaluate if you see possible compartments within the tendon cross‐section to help complement information from displacement tracking and if the tracking reveals slip planes (i.e., locations of shear) presuming subtendon border locations, (5) visually confirm outcomes by evaluating movements from raw videos (e.g., look for slip planes) and compare observations to displacement masks to ensure consistency between masks and regional movement directions. Despite all these efforts, in some cases there was not enough information to create estimates of subtendon locations that filled the entire AT cross‐section, and some areas were left undefined.

**FIGURE 4:**
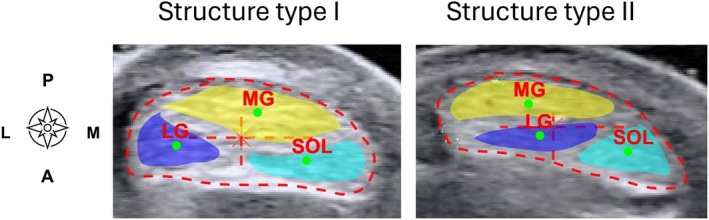
Example of the final output of the analysis. The three subtendon CSAs identified within the entire tendon cross‐section. Geometrical centroids are shown for the entire tendon (large red cross in the mid tendon) and within each subtendon (green dots). Lateral gastrocnemius (LG) shown as blue, medial gastrocnemius (MG) as yellow and soleus (SOL) subtendon as cyan. The examples on left and right were determined as structure type I and II, respectively, based on Pękala et al. [2].

Geometrical centroid (GC) points of each subtendon were identified relative to the centroid of the AT (0.0) using a custom MATLAB script (Figure 4). Then, CSAs of the subtendon and the entire AT were extracted for further analysis. Finally, we compared the identified subtendon locations to the structure types by Pękala et al. [2]. Relative to Pękala et al. [2] our cross‐sections were taken 1 cm proximal to the level of the superior border of the insertion into the calcaneal bone (ICB) but clearly below the muscle‐tendon junction; thus, when deciding the structure type, we weighted the information at ICB.

### 2.5 MRI

Following the protocol and analysis techniques introduced by Cone et al. [12], the AT of two of our participants was imaged using MRI scans performed using a 7‐Tesla MRI Scanner (Siemens Magnetom) and a 28‐channel knee coil with a double echo steady state scan sequence (flip angle: 25°; TR: 25 ms, TE: 2.56 ms; acquisition time: 16 min; FOV:171 × 249 × 90 mm) and a voxel size of 0.558 × 0.558 × 0.4 mm, with no gap between slices. Lying supine with the knee fully extended, scans were performed on the right AT capturing the calcaneus through the gastrocnemius muscle‐tendon junctions with the ankle fixed in a neutral (90°) posture using a custom rigid splint.

Image processing was performed in a commercial software package (Simpleware; Synopsys, USA). An experienced researcher segmented the MG, LG, and SOL subtendons, and 3D models were compiled. GC points of the subtendons relative to the centroid of the AT were calculated from an axial slice with masked subtendons 1 cm above the most proximal calcaneus point, matching the location of USTIM analyses.

### 2.6 Statistical Analysis

Data are presented as means and standard deviations. All statistical tests were performed using JASP (JASP version 0.18, Amsterdam, Netherlands). Intra‐ and inter‐rater (*N* = 12) reliability for data analysis was tested for the entire tendon, MG, LG, and SOL subtendon CSAs, and for the location of their centroids. Both centroid coordinates (X and Y) were included in the analysis as separate data points to yield an intraclass correlation coefficient of [18] and comparison to MRI. Test–retest reliability of the USTIM method was evaluated from three individuals whose tendons were assessed twice on separate days. Linear regression was used to assess the relation between USTIM and MRI subtendon centroid locations and CSAs of the subtendons. The coefficient of determination (*r* ^2^) was calculated for each variable and interpreted as follows: little or no relation (0.00–0.19), fair (0.20–0.49), moderate to good (0.50–0.69), or strong (≥ 0.70) [20, 21].

## 3 Results

Of the 14 participants measured, four had considerable involuntary leg movement in response to stimulation. When leg movement was present, the data could not be appropriately analyzed, so only trials free from leg movement were included in the analysis. A total of 12 legs from 10 participants were included in the final sample (both legs were included for 2 participants).

### 3.1 Relative Subtendon Sizes and Locations

**FIGURE 5:**
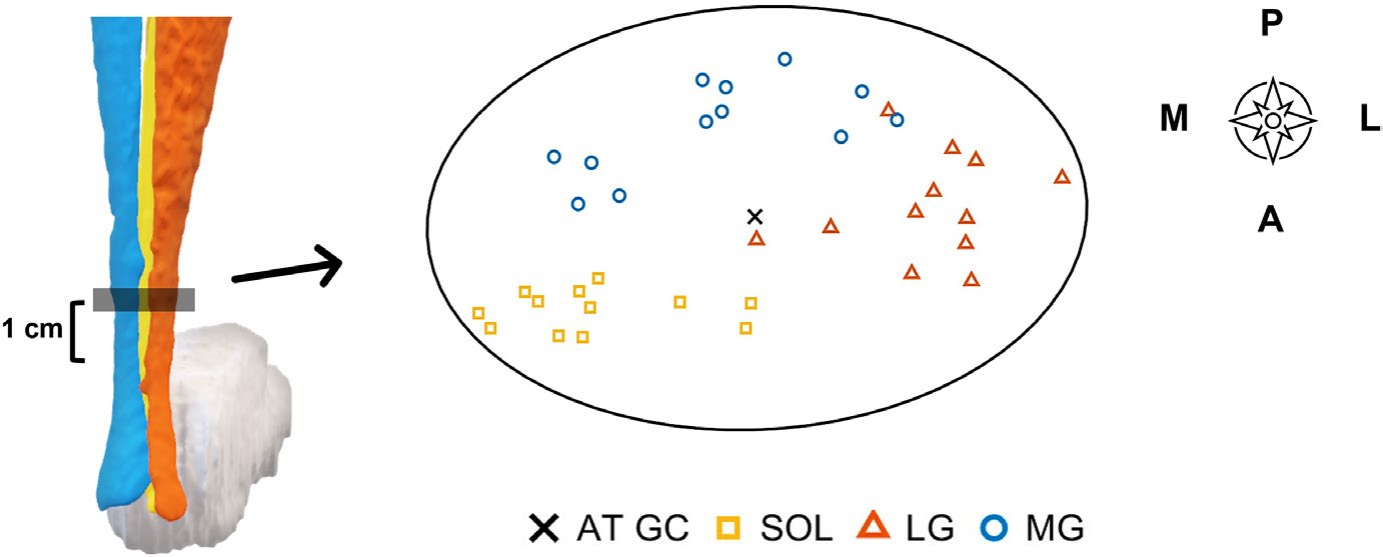
Representation (*N* = 12) of the identified subtendon centroid locations within the AT cross‐section at 1 cm above the most proximal calcaneal point.

In most of our sample, MG was located in the postero‐medial part of the tendon, LG in the antero‐lateral part, and SOL occupied the antero‐medial portion of the tendon (Figures 3 and 5). The subtendon CSA estimated 1 cm proximal to the most proximal calcaneal echo signal was on average (mean ± SD) 0.20 ± 0.06 cm^2^ for the MG, 0.14 ± 0.03 cm^2^ for LG, and 0.13 ± 0.04 cm^2^ for the SOL muscle. These represented on average 35% (MG), 24% (LG), and 22% (SOL) of the whole tendon cross‐sectional area (0.59 ± 0.11 cm^2^). Approximately 19% of the tendon area was left undefined.

Based on the twist classification of Pękala et al. [2] the 12 tendons were divided into different twist groups according to the subtendon locations at the level of the superior insertion into the calcaneal point [2]. Of the 12 tendons (10 individuals) analyzed, 83% (80%) were identified as type I, and 17% (20%) as type II/III.

### 3.2 Reliability

#### 3.2.1 Inter‐and Intra‐Rater Reliability

Intra‐rater ICC_3ˌ1_ for the subtendon centroid locations was 0.79 (95% CI 0.70–0.85) and 0.82 (95% CI 0.75–0.87) for the two independent raters. ICC_3ˌ1_ for subtendon CSA was 0.70 (95% CI 0.60–0.79) and 0.79 (95% CI 0.71–0.85). For the entire tendon CSA, intra‐rater ICC_3ˌ1_ for the two independent raters was 0.96 (95% CI 0.94–0.97) and 0.83 (95% CI 0.77–0.88).

Inter‐rater reliability for the subtendon centroid locations ICC_1ˌ1_ was 0.77 (95% CI 0.69–0.84). ICC_1ˌ1_ for the subtendon CSA's was 0.53 (95% CI 0.39–0.65) and 0.81 (95% CI 0.74–0.87) for the entire AT CSA.

#### 3.2.2 Test–Retest Reliability

The USTIM method test–retest reliability ICC_3ˌ1_ from participants assessed on different days was 0.93 (95% CI 0.90–0.88) for subtendon centroid locations, 0.88 (95% CI 0.83–0.92) for subtendon CSAs, and 0.97 (95% CI 0.97–0.98) for the entire tendon area.

#### 3.2.3 Validation of USTIM Method Versus MRI

**FIGURE 6:**
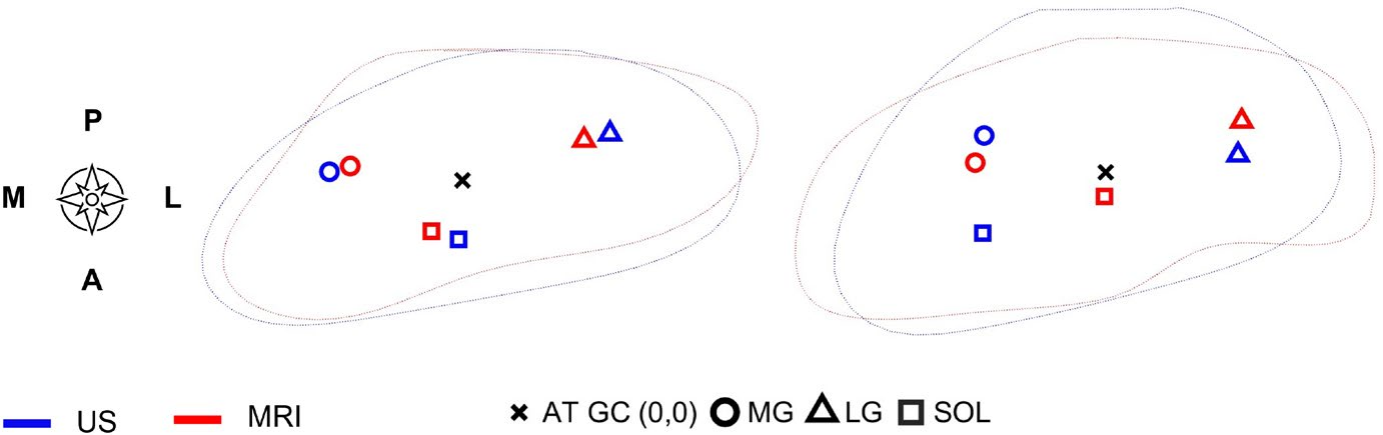
Subtendon centroid locations identified using the USTIM method versus MRI in two different individuals (left and right). The slices were oriented based on the tendon geometrical centroid (AT GC).

A strong relationship was found between the USTIM method and MRI‐based subtendon centroid locations (Figure 6) (*r* ^2^ = 0.80, *F*(1,11) = 46.3, *p* < 0.001) and moderate‐to‐good for the subtendon CSAs (*r* ^2^ = 0.63, *F*(1,5) = 6.7, *p* = 0.060).

## 4 Discussion

We showed that a novel USTIM method can identify the locations of the three AT subtendons in vivo in humans. The method, combining ultrasonography and electrical stimulation, provided complementary results to high‐field MRI and showed moderate to excellent reliability. In our sample of 10 individuals, we identified 8 individuals with type I tendons and 2 individuals with type II/III tendons according to the structure types presented by Pękala et al. [2].

There was a strong association between *subtendon centroid locations* identified using the USTIM method and 7 T MRI. The largest difference in centroid locations was found in the SOL of one participant. Although knee and ankle positions (0° and 90°) were similar between the two methods, slight differences in the orientation of the AT were present and may partly contribute to the differences observed in Figure 6. Despite the slight differences, our results provide evidence of the utility of the USTIM method to identify the locations of the subtendons within the AT. Subtendon CSAs were consistently greater for MRI than the USTIM method (Table 1). This may partially arise from the fact that with USTIM, not all AT area could be allocated to subtendons, and further efforts are needed to achieve consistent decision‐making of the areas.

*Subtendon CSAs* estimated using USTIM showed a moderate to good association with MRI. Our method systematically underestimated the areas of the subtendons compared to MRI. In ultrasound images, we followed the inner border of the AT when drawing the ROI to avoid possible sliding movement in the peritendon area. Identifying sizes of the subtendons was challenging for several reasons. First, the connections between the subtendons are tighter in the distal region [22], where we performed the imaging. It may be that in some cases, these tight connections made it challenging to identify movement in a distinct area. Furthermore, low stimulation intensity might not cause displacement of all fascicles within a subtendon. Hence, identification of a subtendon location was possible with low thresholds but may not reflect the entire subtendon area. On the other hand, higher stimulation intensity can cause force sharing between the subtendons, causing the detected movement area to be greater than the area of the targeted subtendon. Furthermore, stimulation may have induced lateral force transmission to adjacent muscle and/or subtendon, causing overlapping regions to be identified in response to stimulation of different muscles.

Stimulation intensity should be optimized to generate subtendon twitch without transferring movement to neighboring tissues. Lastly, movement might occur in the interfascicular matrix without a change in the length of the tendon fibers, suggesting that the movement might occur in the intra‐tendinous fascicular matrix and not in the subtendon fascicles [23, p2]. These factors may collectively explain why on average 19% of the total AT CSA was unassigned to any subtendon.

Test–retest reliability of the USTIM method between 2 days was found to be excellent for identifying subtendon centroids and good for identifying subtendon area. Reliability within and between raters in identifying the subtendon centroids was found to be good. Reliability for identifying subtendon area was good for test–retest and intra‐raters but moderate between raters. Improving reliability is recognized as a point for methodological improvement since the current approach relies partly on subjective decision‐making. The current study was performed using a standard desktop with a computer mouse, but the method may be more efficient and potentially more precise through the use of specialized image processing tools (i.e., stylus and drawing pad equipment). While using high‐field MRI may provide a comprehensive reconstruction of the tendon, the USTIM method can give important subject‐specific information at lower cost and in a more accessible manner. In the future, adding more imaging planes would provide a viable method for imaging the locations of the subtendon across the length of the tendon, allowing three‐dimensional reconstruction similar to MRI.

The presented USTIM method revealed tendon structure types that align with previous cadaveric studies [1, 2, 3]. Most of the sample was classified as type I, with 2 individuals out of the 10 measured participants classified as type II (Table 2). In our cohort, a relatively higher percentage (83%) were identified as type I compared to prior cadaveric studies that categorized 48% [2] and 50% [1] as type I. Our results align with Cone et al. [12] that utilized high field 7 T MRI to unravel the substructure of the AT and identified 80% of the tendons as type I. The difference to previous studies may be attributed to the nature of the in vivo analysis compared to cadaveric studies, in addition to the participant population. It is important to note that most participants had non‐twisted tendons. Those with more twisted tendons could experience greater internal compression and intratendinous pressure than tendons with less twist [24]. This suggests that a highly twisted tendon structure might be a risk factor for AT injury [24, 25]. Identifying different subject‐specific structure types could be valuable in clinical settings for developing prevention strategies and individualized rehabilitation protocols.

**TABLE 2:** Achilles tendon cross‐sectional area, subtendon length, and twist types classified according to Pękala et al. [2]. Of the 10 individuals, 80% had twist type I, or if calculated using number of legs analyzed, 83% of the legs were type I.

Participant number (leg)	Tendon cross‐sectional area (cm^2^)	MG subtendon length (cm)	LG subtendon length (cm)	SOL subtendon length (cm)	Twist type	
					Rater1	Rater2
1	0.74	16.7	19.4	4.6	I	I
2/R	0.67	19	21.3	4.2	I	I
2/L	0.80	17.5	21.7	4.3	I	I
3	0.42	20.8	22	5.7	I	I
4	0.70	20.5	21.4	5.4	I	I
5	0.54	17.2	19.1	4.6	II/III	II
6	0.48	16.3	17.6	4.2	I	I
7	0.59	14.9	19.3	3.9	I	I
8/R	0.56	18.8	21.7	3.9	I	I
8/L	0.59	16.9	21.8	3.1	I	I
9	0.59	22.4	25.8	5.4	II/III	II
10	0.50	15	18	5	I	I

**TABLE 1:** Subtendon CSAs estimated with US and MRI.

Participant	MG		LG		SOL	
	1	2	1	2	1	2
MRI (cm^2^)	0.32	0.24	0.29	0.35	0.17	0.21
USTIM (cm^2^)	0.23	0.17	0.18	0.18	0.14	0.16

Similar to cadaveric and in vivo studies, we found individual variation in the subtendon locations, geometry, and CSA of the AT (Figure 5). We found that MG occupies the largest portion of the AT with 35%, followed by LG 24%, and SOL 22%, estimated 1 cm above the most proximal point of the calcaneus. It is to be noted that our method revealed the subtendon CSAs only at the assessed plane and did not cover the entire cross‐section across the length of the tendon. Cone et al. [12] reported average subtendon CSAs across the length of the tendon as follows: MG 37%, LG 41%, and SOL 22%. If we take values reported by Cone et al. [12] approximately 1 cm proximal to the calcaneal insertion, we find that MG occupied roughly 40%, the LG roughly 40%, and the SOL roughly 20% of the AT cross‐section, respectively, following a similar pattern to our findings. In contrast, Klaiber et al. [14] placed the USG probe more proximally and found that MG (30%) covered twice the area of LG (15%), and the authors proposed that the remaining portion of the tendon is occupied by the SOL subtendon, accounting for 55% of the tendon CSA. At the level of the insertion into the calcaneal bone, Pękala et al. [2] found that 44% of the tendon was occupied by LG, 28% by MG, and 28% by SOL. Further, in cadavers, in the thinnest region about 1–4 cm proximal to calcaneal insertion, the SOL subtendon has been reported to cover on average 59% of the total area [26]. Heterogeneous results between studies can be partly explained by the larger inter‐individual variation of the AT substructure [2, 12], and the variation of the physiological cross‐sectional area (PCSA) ratio between the triceps surae (TS) muscles [27, 28].

Is it realistic that the SOL muscle, which has a PCSA nearly five times greater than the LG and three times greater than that of the MG, is capable of generating the biggest amount of force among the TS muscles [27, 29], and would have a subtendon accounting for only about 22% of the total AT area? Tendon material properties vary among different muscles sometimes counterintuitively; in turkeys, Matson et al. [30] showed greater modulus in the small flexor hallucis longus muscle compared to the gastrocnemius muscle. In humans, the SOL subtendon has a lower tensile modulus compared to the MG subtendon [23] while the SOL muscle has a lower rate of force development and contains more slow‐type muscle fibers compared to the gastrocnemius [29, 31, 32]. Future studies should investigate differences in material properties of the AT subtendons to confirm and better understand these in vivo functional observations.

### 4.1 Limitations

Our novel USTIM method relies on the assumption that the first movement observed in tendon tissue is anatomically associated with the stimulated muscle. Due to the existence of physical connections between adjacent TS muscles [22] there is a possibility of lateral force transmission between the adjacent tissues both at the muscle and tendon levels, which could have influenced the identification of subtendon CSAs. While force transmission between tendon fascicles can be negligible at low forces [33], lateral force transmission does occur at higher stimulation intensities [16, 34]. Therefore, low stimulation intensity was used to ensure isolated activation of the targeted muscle.

## 5 Conclusion

We successfully used a novel USTIM method combining ultrasonography and electrical stimulation to identify the locations of the three AT subtendons in humans in vivo. The method was shown to be feasible, with results comparable to high‐field MRI and moderate‐to‐excellent reliability. This method may be used to better understand the intricacies of structure–function relationships in the Achilles tendon. Knowledge of subtendon locations may contribute to understanding the etiology of different pathologies and facilitating personalized training and rehabilitation interventions.

### 5.1 Perspective

Recent research highlights the impact of Achilles tendon twisting degree on strain, compression, and intra‐tendinous pressure [24, 25]. Utilizing the novel USTIM method, we can identify the in vivo locations of the three subtendons within the cross‐section of the human AT. This advancement would not only enhance our understanding of the complex structure –function relationships of the AT but also hold potential clinical value. Knowledge about individual subtendon locations can be valuable for developing targeted prevention strategies, better surgical techniques, and personalized rehabilitation protocols.

## Author Contributions

**Taija Finni:** conception and design, acquisition of data, analysis, interpretation of data, drafting the manuscript, reviewing and editing, and approving the final version. **Raad Khair:** conception and design, acquisition of data, analysis, interpretation of data, drafting the manuscript, reviewing and editing, and approving the final version. **Jason R. Franz:** acquisition of data, reviewing and editing, and approving the final version. **Maria Sukanen:** analysis, reviewing and editing, and approving the final version. **Neil Cronin:** analysis, reviewing and editing, approving the final version. **Stephanie Cone:** acquisition of data, analysis, reviewing and editing, and approving the final version.

## Ethics Statement

The study received approval from the Human Research Ethics Committee of the University of Jyväskylä on 21.12.2022 (1351/13.00.04.00/2022). The MR images were collected following informed consent as per the UNC‐CH Biomedical Sciences Institutional Review Board (IRB # 21‐0059).

## Conflicts of Interest

Two of the authors (T.F. and R.K.) are co‐inventors on a pending patent application on the USTIM technology described herein. Other authors declare no conflicts of interest.

## Supporting information


Video S1.

Video S2.

Video S3.

Appendix S1.

## Data Availability

The data that support the findings of this study are available on request from the corresponding author. The data are not publicly available due to privacy or ethical restrictions.
