# Supplementary material for: A Novel Method to Assess Subject‐Specific Architecture of the Achilles Tendon In Vivo in Humans
Source: Scand J Med Sci Sports. 2025 Mar 26;35(4):e70042. doi: 10.1111/sms.70042 (PMC11938201; doi:10.1111/sms.70042)

# Supplement 1

# Analyses from the videos S2-S4

The below image is a screenshot from the videos. The Achilles tendon is localized in the middle of the video.


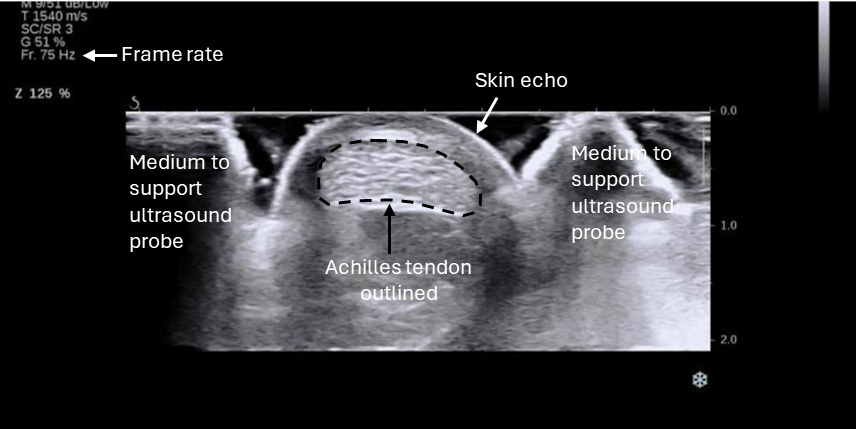


From each muscle stimulation video, the first and second frames showing initial movement, marked as green, can be found below. Green areas show where the pixel intensities have changed in response to selective muscle stimulation of either soleus (SOL, S2), lateral (LG, S3) or medial gastrocnemius muscle (MG, S4). It is to be acknowledged that the regions are not always distinct, which may be due to some lateral force transmission at the muscle and/or tendon.

**S2_videoSOLstim.avi**

In the below images (1 and 2), SOLstim video was analysed with threshold 9.


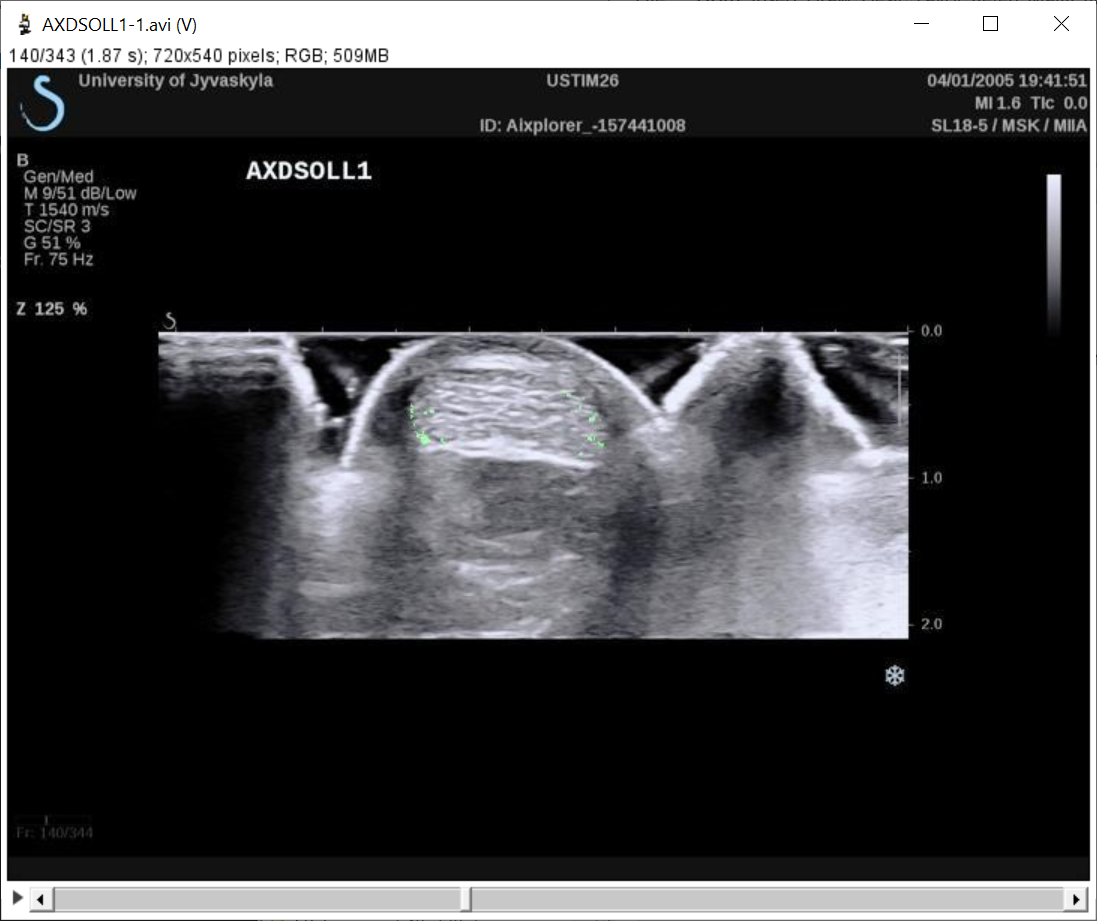


Image 1: 1^st^ frame: movement occurs close to borders on both sides (nearly similarly as in LGstim condition).


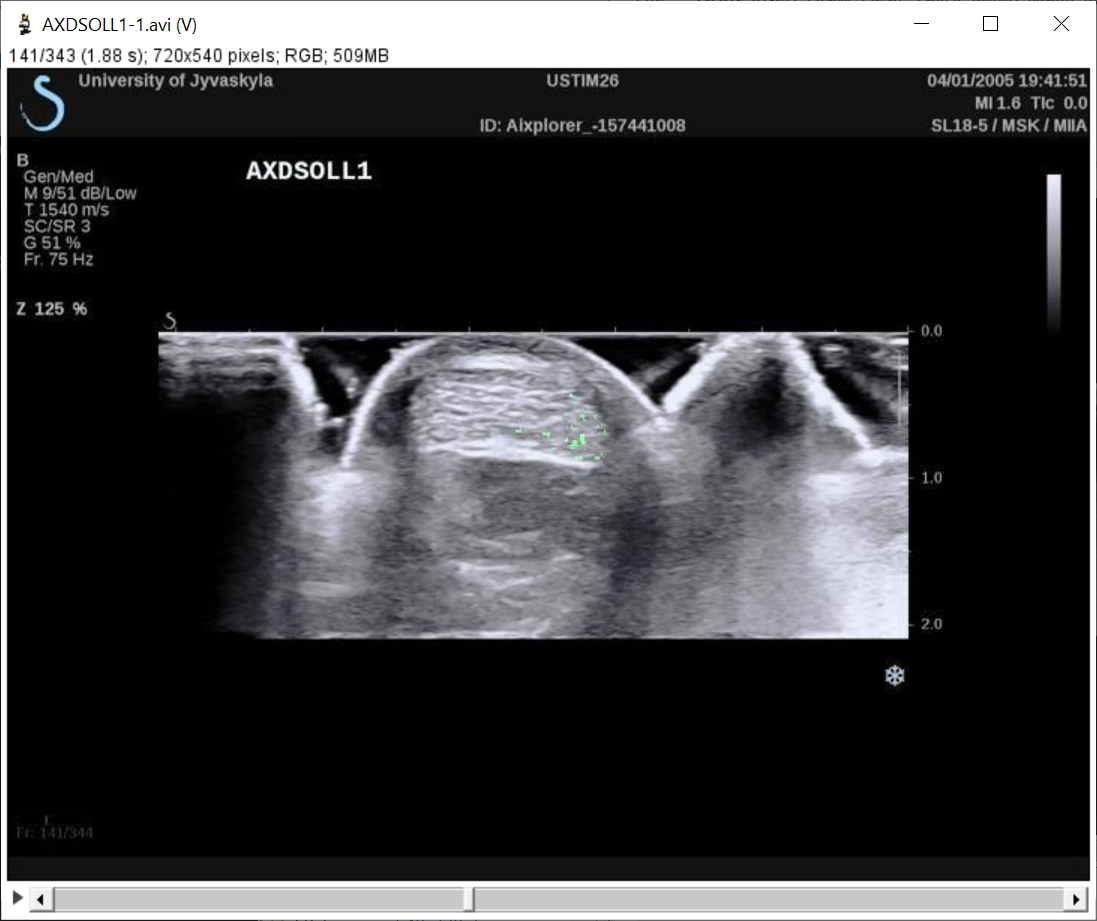


Image 2: 2^nd^ frame: movement is localized to a lower right-hand corner. One may also distinguish compartment on the region that has slightly lower echointensity than the remaining tendon. Thus, SOL location was identified to be in the area with green highlights. This example corresponds to that in Figure 3B. The green areas are slightly different, since we typically used several thresholds in the analysis to confirm the locations.

**S3_videoLGstim.avi**

In the below images (3 and 4), LGstim video was analysed with threshold 7. The first and second frames showing movement are displayed.


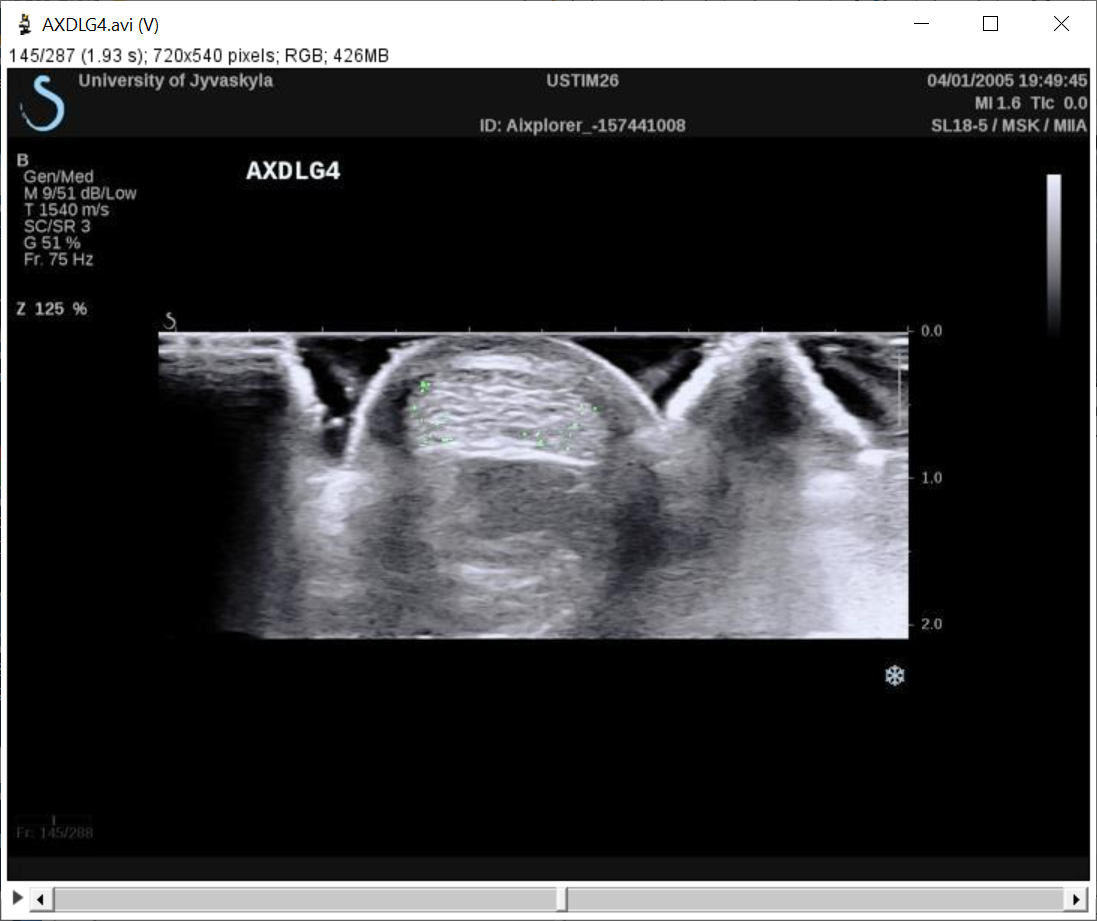


Image 3: 1^st^ frame: movement occurs close to borders on both sides (similarly as in SOLstim condition).


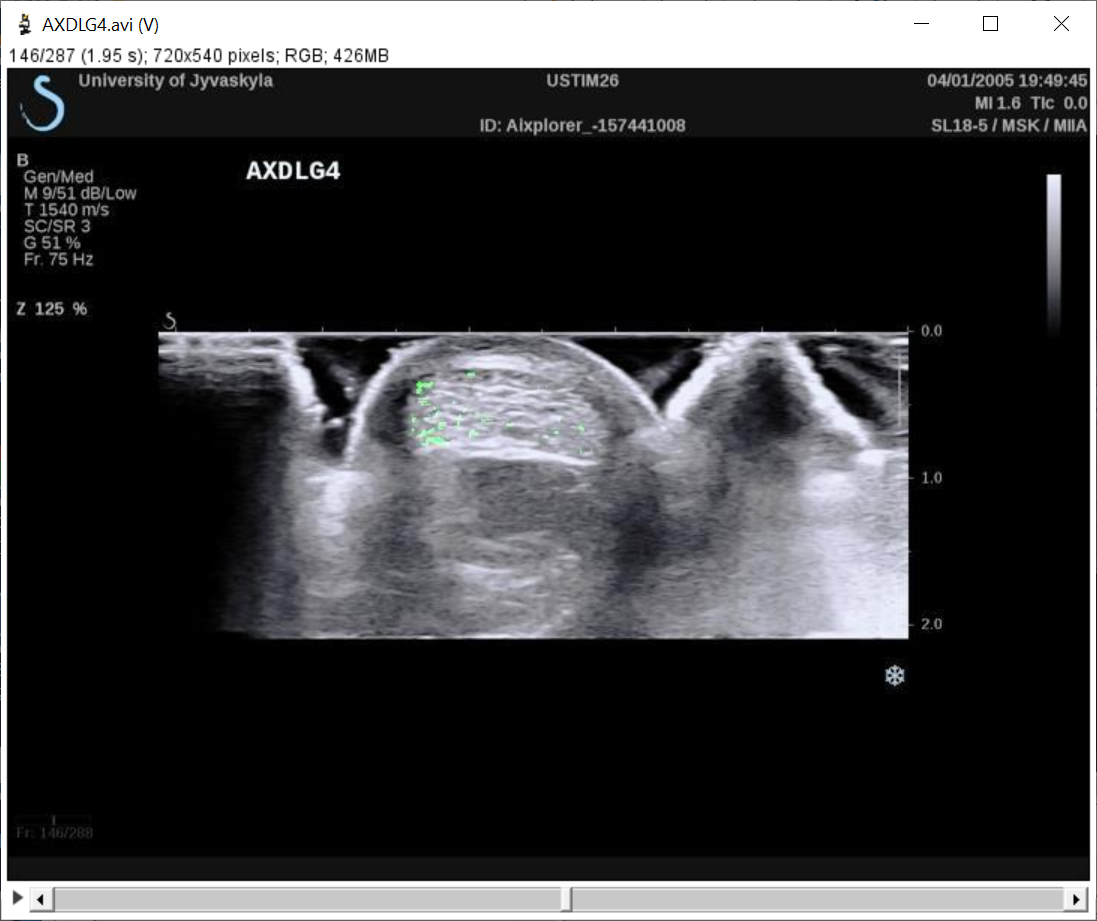


Image 4: 2^nd^ frame: Most movement occurs on the left side, which is the presumed LG subtendon.

**S4_videoMGstim.avi**

In the below images (5 and 6), MGstim video was analysed with threshold 7. The first and second frames showing movement are displayed.


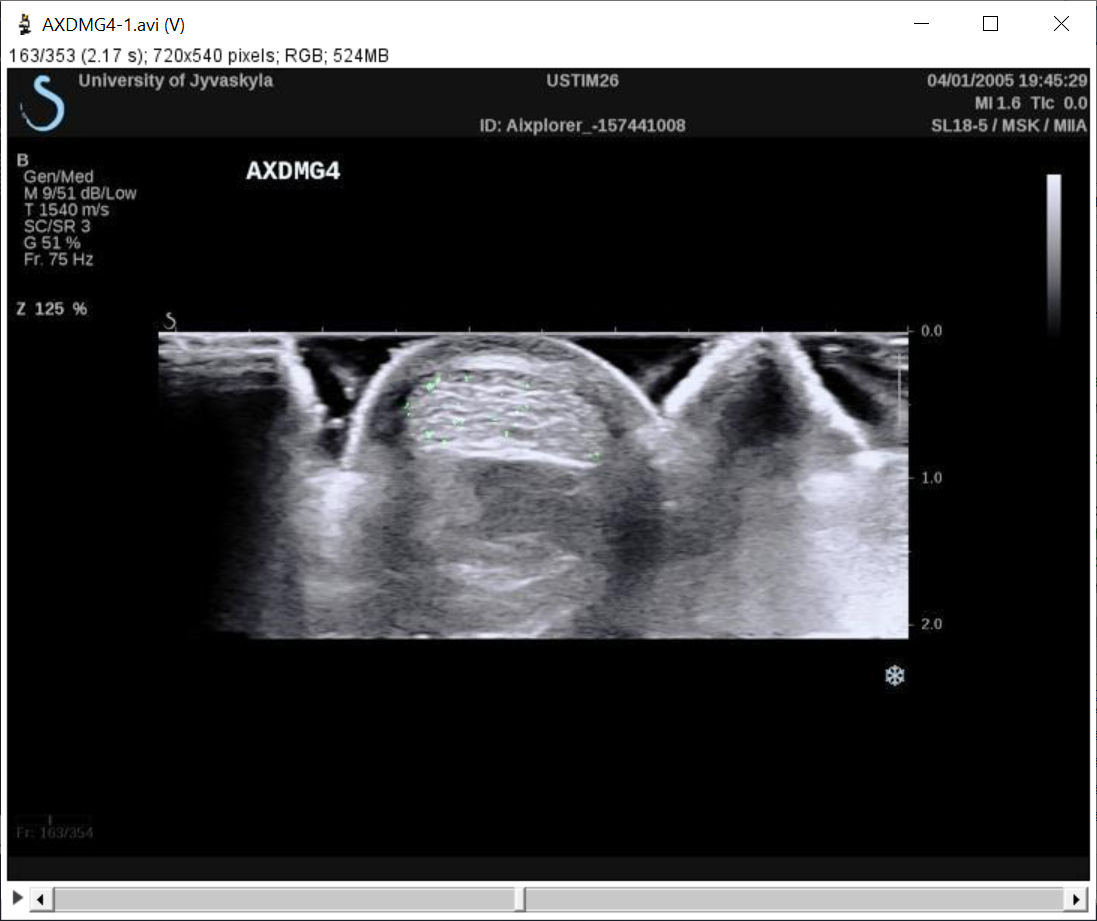


Image 5: 1^st^ frame. Small movements occur around larger region.


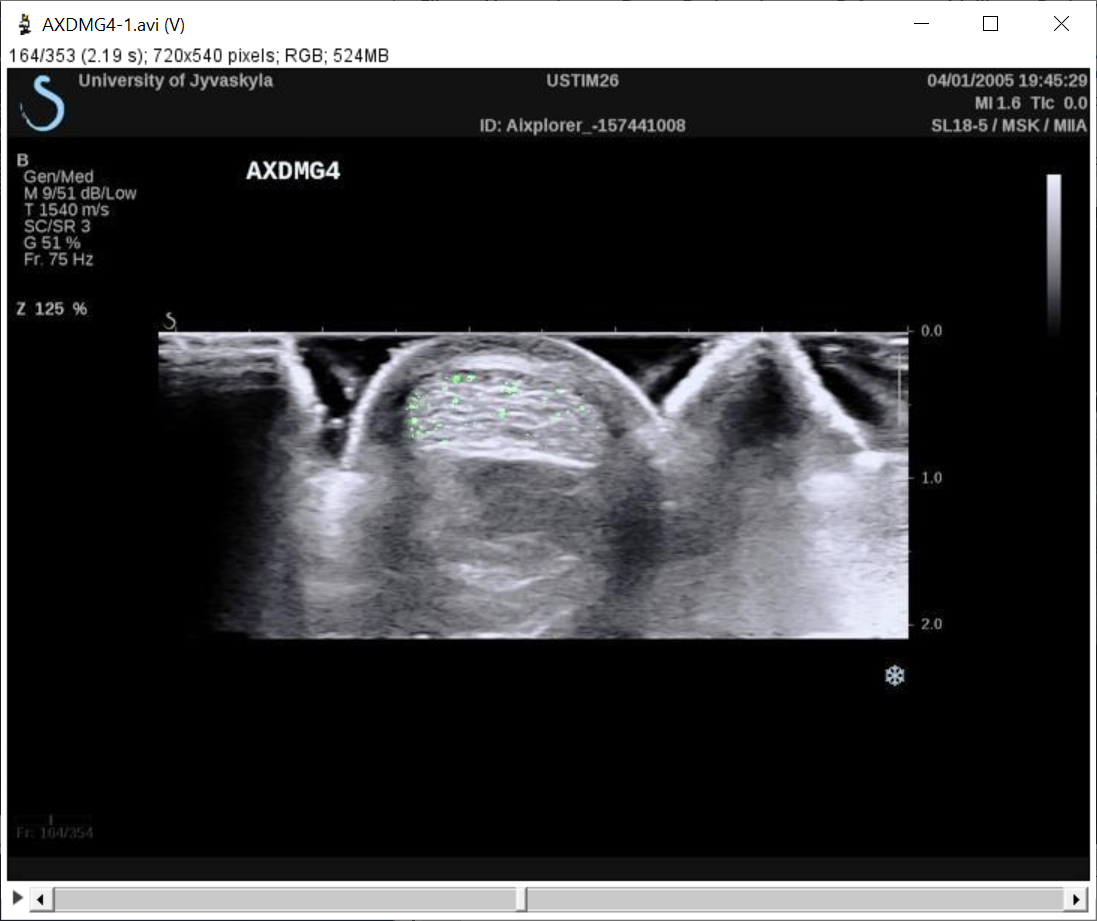


Image 6: 2^nd^ frame. Movement is not occurring in the defined SOL area but overlaps with presumed LG region. Subtraction logic is used and the MG subtendon defined to locate in the middle posterior region. Because LG stimulation produced a distinct region, that location is omitted and deduced that MG must lay on the area remaining after SOL and LG have been identified.

The decision-making process involved reviewing the analyzed videos several times and often analyzing with different thresholds to confirm consistency. Videos were also analyzed with different stimulation intensities, but often the lowest intensity providing movement was most selective in showing local displacements. Sometimes examination of the raw videos was helpful to confirm visually patterns of displacement. For example, in the MGstim video, the posterior region in the top of the image shows distinct movement whereas in other videos the movement pattern is very different.

The final result:


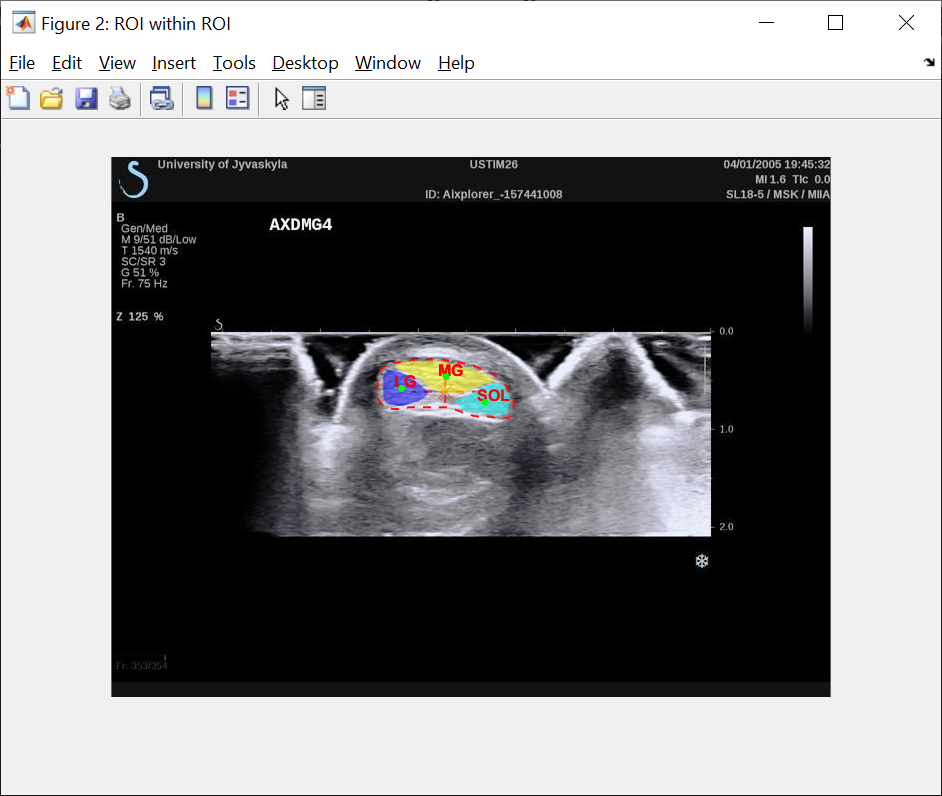

Supplement: Supplementary file 4 — Appendix S1. [file SMS-35-e70042-s001.doc]
